# Supplementary material for: Controllable strain-driven topological phase transition and dominant surface-state transport in HfTe5
Source: Nat Commun. 2024 Jan 6;15:332. doi: 10.1038/s41467-023-44547-7 (PMC10771548; doi:10.1038/s41467-023-44547-7)
Supplement: Supplementary file 1 — Supplementary Information file [file 41467_2023_44547_MOESM1_ESM.pdf]

Supplementary Information for Controllable strain-driven topological phase transition and dominant surface-state transport in $HfTe_5$

Jinyu Liu[1], Yinong Zhou[1], Sebastian Yepez Rodriguez[1], Matthew A. Delmont[2], Robert A. Welser[1], Triet Ho[2], Nicholas Sirica[3], Kaleb McClure[4], Paolo Vilmercati[4], Joseph W. Ziller[5], Norman Mannella[4], Javier D. Sanchez-Yamagishi[1], Michael T. Pettes[3], Ruqian Wu[1], Luis A. Jauregui[1,*]

[1]Department of Physics and Astronomy, University of California, Irvine, CA 92697, USA

[2]Department of Mechanical and Aerospace Engineering, University of California, Irvine, CA 92697, USA

[3]Center for Integrated Nanotechnologies (CINT), Materials Physics and Applications Division, Los Alamos National Laboratory, Los Alamos, NM 87544, USA

[4]Department of Physics and Astronomy, The University of Tennessee, Knoxville, TN, 37996, USA

[5]Department of Chemistry, University of California, Irvine, CA 92697, USA

Contents

I. **Single crystal X-ray diffraction on a CVT-grown $HfTe_5$ crystal**

II. **Fermi surface and constant energy contour measured from ARPES**

III. **Magnetoresistance characterization of a free-standing $HfTe_5$ sample**

IV. **Comparison of different functionals for DFT calculations**

V. **Band structure of $HfTe_5$ at zero strain**

VI. **Wannier charge centers and $\mathbb{Z}_2$ indices under different strains**

VII. **Band structures for various cases of strain along the *c* axis**

VIII. **Application of small strain with the home-built single piezo-stack strain cell**

IX. **Application of large strain with the home-built bending strain cell**

I. Single crystal X-ray diffraction on a CVT-grown $HfTe_5$ crystal

Single crystal X-ray diffraction was performed under liquid nitrogen flow on a piece of $HfTe_5$ single crystal (grown by CVT). A small crystal with dimensions approximately 0.037 mm × 0.157 mm × 0.270 mm was mounted in a cryoloop and transferred to a Bruker SMART APEX II diffractometer system. The APEX2[1] program package was used to determine the unit-cell parameters and for data collection (10 sec/frame scan time). The raw frame data was processed using SAINT[2] and SADABS[3] to yield the reflection data file. Subsequent calculations were carried out using the SHELXTL[4] program package. The diffraction symmetry was mmm and the systematic absences were consistent with the orthorhombic space group Cmcm. The structure was solved by direct methods and refined on F^2 by full-matrix least-squares techniques. The analytical scattering factors[5] for neutral atoms were used throughout the analysis. Least-squares analysis yielded wR2 = 0.0521 and Goof = 1.328 for 22 variables refined against 753 data (0.68 Å), R1 = 0.0200 for those 749 data with $I > 2.0\sigma(I)$. Three sets of diffraction data were collected at three different temperatures (T = 93K, 173K, and 223K).

Definitions for refinement: $wR2 = [\Sigma[w(F_o^2-F_c^2)^2] / \Sigma[w(F_o^2)^2]]^{½}$; $R1 = \Sigma||F_o|-|F_c|| / \Sigma|F_o|$; Goof = $S = [\Sigma[w(F_o^2-F_c^2)^2] / (n-p)]^{1/2}$ where n is the number of reflections and p is the total number of parameters refined. The thermal ellipsoid plot is shown at the 50% probability level.

The crystal structure is confirmed to be orthorhombic with a space group of *Cmcm*, as reported in literature[6]. The refinement result including lattice constants and atomic coordinates is used as the initial input for the first-principles calculations and ARPES data analysis. The lattice constants obtained from the diffraction measurements done at various temperatures at 93 K, 173 K, and 223 K show a general trend of contraction with cooling.

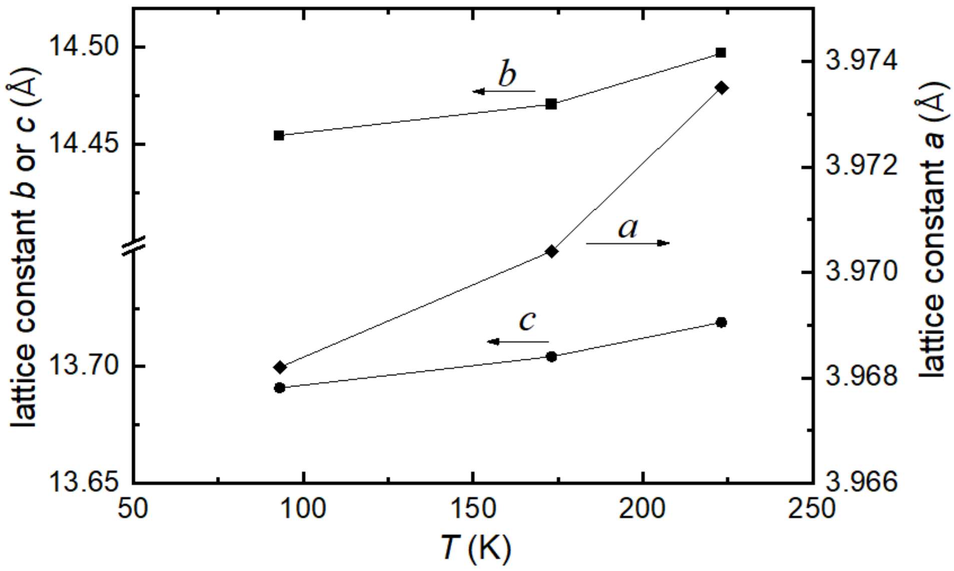

Supplementary Figure 1. Lattice constants obtained from the single crystal X-ray diffraction at multiple temperatures.

Supplementary Table 1. Crystal data and structure refinement for a $HfTe_5$ crystal (T = 93 K).

Identification code	HfTe5	
Empirical formula	$[Hf\ Te_5]_\infty$	
Formula weight	816.49	
Temperature	93(2) K	
Wavelength	0.71073 Å	
Crystal system	Orthorhombic	
Space group	*Cmcm*	
Unit cell dimensions	a = 3.9682(5) Å	a = 90°.
	b = 14.4548(17) Å	b = 90°.
	c = 13.6909(16) Å	γ = 90°.
Volume	785.30(16) $Å^3$	
Z	4	
Density (calculated)	6.906 Mg/m^3	
Absorption coefficient	31.376 mm^{-1}	
F(000)	1328	
Crystal color	black	
Crystal size	0.270 x 0.157 x 0.037 mm^3	
Theta range for data collection	2.818 to 31.614°	
Index ranges	$-5 \leq h \leq 5$, $-20 \leq k \leq 20$, $-20 \leq l \leq 19$	
Reflections collected	9750	
Independent reflections	753 [R(int) = 0.0322]	
Completeness to theta = 25.242°	100.0 %	
Absorption correction	Semi-empirical from equivalents	
Max. and min. transmission	0.1017 and 0.0258	
Refinement method	Full-matrix least-squares on F^2	
Data / restraints / parameters	753 / 0 / 22	
Goodness-of-fit on F2	1.328	
Final R indices [I > 2sigma(I) = 749 data]	R1 = 0.0200, wR2 = 0.0521	
R indices (all data, ? Å)	R1 = 0.0202, wR2 = 0.0521	
Extinction coefficient	0.00096(7)	
Largest diff. peak and hole	1.987 and -1.351 e.$Å^{-3}$	

Supplementary Table 2. Atomic coordinates ($\times 10^4$) and equivalent isotropic displacement parameters ($Å^2 \times 10^3$)
for the $HfTe_5$ sample (T = 93 K). U(eq) is defined as one-third of the trace of the orthogonalized U^{ij} tensor.

	x	y	z	U(eq)
Hf(1)	5000	6851(1)	2500	9(1)
Te(1)	5000	7902(1)	4354(1)	9(1)
Te(2)	0	5696(1)	1494(1)	10(1)
Te(3)	0	8366(1)	2500	9(1)

Supplementary Table 3. Bond lengths [Å] and angles [°] for the $HfTe_5$ sample (T = 93 K).

Hf(1)-Te(2)	2.9363(4)
Hf(1)-Te(2)#1	2.9363(4)
Hf(1)-Te(2)#2	2.9363(4)
Hf(1)-Te(2)#3	2.9363(4)
Hf(1)-Te(3)	2.9553(5)
Hf(1)-Te(3)#3	2.9553(5)
Hf(1)-Te(1)	2.9575(5)
Hf(1)-Te(1)#1	2.9576(5)
Te(1)-Te(1)#4	2.9022(6)
Te(1)-Te(1)#5	2.9022(6)
Te(2)-Te(2)#1	2.7551(9)
Te(2)-Hf(1)#6	2.9363(4)
Te(3)-Hf(1)#6	2.9553(5)
Te(2)-Hf(1)-Te(2)#1	55.958(17)
Te(2)-Hf(1)-Te(2)#2	110.69(2)
Te(2)#1-Hf(1)-Te(2)#2	85.020(15)
Te(2)-Hf(1)-Te(2)#3	85.020(15)
Te(2)#1-Hf(1)-Te(2)#3	110.69(2)
Te(2)#2-Hf(1)-Te(2)#3	55.957(17)
Te(2)-Hf(1)-Te(3)	88.151(12)
Te(2)#1-Hf(1)-Te(3)	88.152(12)
Te(2)#2-Hf(1)-Te(3)	151.052(10)
Te(2)#3-Hf(1)-Te(3)	151.052(10)
Te(2)-Hf(1)-Te(3)#3	151.052(10)
Te(2)#1-Hf(1)-Te(3)#3	151.052(10)
Te(2)#2-Hf(1)-Te(3)#3	88.152(12)
Te(2)#3-Hf(1)-Te(3)#3	88.152(12)
Te(3)-Hf(1)-Te(3)#3	84.346(19)
Te(2)-Hf(1)-Te(1)	133.995(8)
Te(2)#1-Hf(1)-Te(1)	83.659(11)
Te(2)#2-Hf(1)-Te(1)	83.659(12)
Te(2)#3-Hf(1)-Te(1)	133.995(8)
Te(3)-Hf(1)-Te(1)	67.624(9)
Te(3)#3-Hf(1)-Te(1)	67.624(9)
Te(2)-Hf(1)-Te(1)#1	83.658(12)
Te(2)#1-Hf(1)-Te(1)#1	133.995(8)
Te(2)#2-Hf(1)-Te(1)#1	133.995(8)
Te(2)#3-Hf(1)-Te(1)#1	83.659(12)
Te(3)-Hf(1)-Te(1)#1	67.624(9)
Te(3)#3-Hf(1)-Te(1)#1	67.623(9)
Te(1)-Hf(1)-Te(1)#1	118.18(2)
Te(1)#4-Te(1)-Te(1)#5	86.26(2)
Te(1)#4-Te(1)-Hf(1)	108.516(19)
Te(1)#5-Te(1)-Hf(1)	108.52(2)
Te(2)#1-Te(2)-Hf(1)#6	62.021(9)
Te(2)#1-Te(2)-Hf(1)	62.021(9)
Hf(1)#6-Te(2)-Hf(1)	85.020(16)
Hf(1)-Te(3)-Hf(1)#6	84.346(19)

II. Fermi surface and constant energy contour measured from ARPES

Determination of high symmetry axes, k_c and k_a, was performed by measuring the Fermi surface of $HfTe_5$ following sample cleave. Here, $\Delta\theta$ = 1.0° polar angle steps were taken over a -17° < θ < 17° range, spanning -0.6 Å^{-1} < k_a < 0.6 Å^{-1} along the $X\Gamma X$ direction (Supplementary Figure 2). For the analyzer slit oriented along YΓY, both the Fermi surface (Supplementary Figure 2a)) and constant energy contour obtained at E_{BE} = 0.1 eV (Supplementary Figure 2b) illustrate the opening of a hole pocket at G within both the first and second surface Brillouin zone of the (010) face. Such energy contours were constructed following a +/- 12.5 meV (2×ΔE) integration about E_F and E_{BE} = 0.1 eV respectively, where the tail of the valence band maximum is found to cross the Fermi level at T = 77 K (Supplementary Figure 2c).

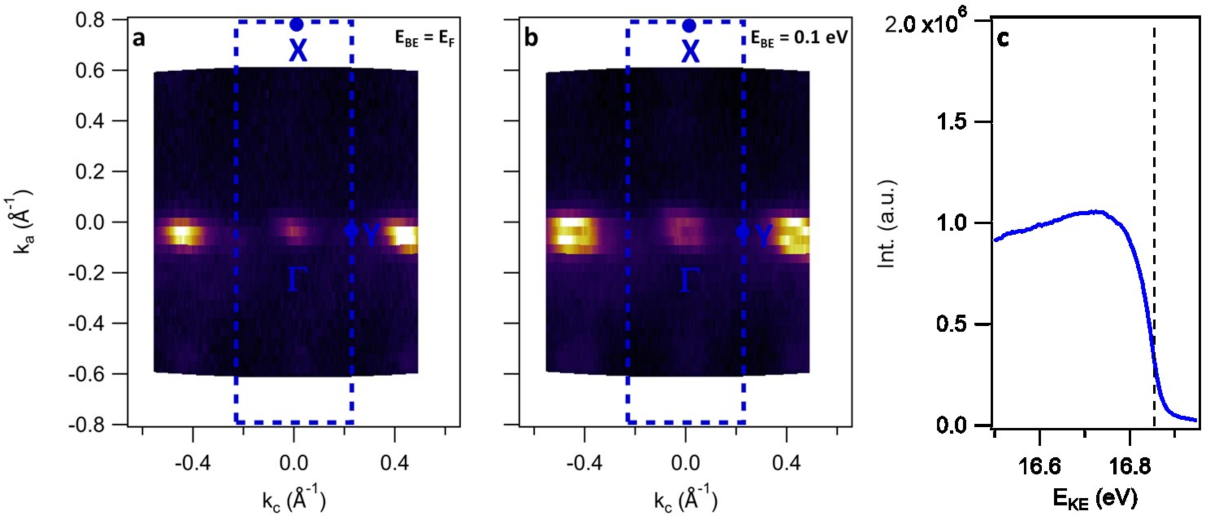

Supplementary Figure 2. a, Fermi surface and **b,** constant energy contour (E_{BE} = 0.1 eV) measured from (010) cleaved $HfTe_5$. The surface Brillouin zone is shown as a blue dashed line, with high symmetry points Γ, X, and Y labeled accordingly. **c**, Integrated energy distribution curve along the YΓY cut illustrating the position of the valence band maximum with respect to Fermi level (dashed) calibrated in photoelectron kinetic energy (E_{KE}).

III. Magnetoresistance characterization of a free-standing $HfTe_5$ sample

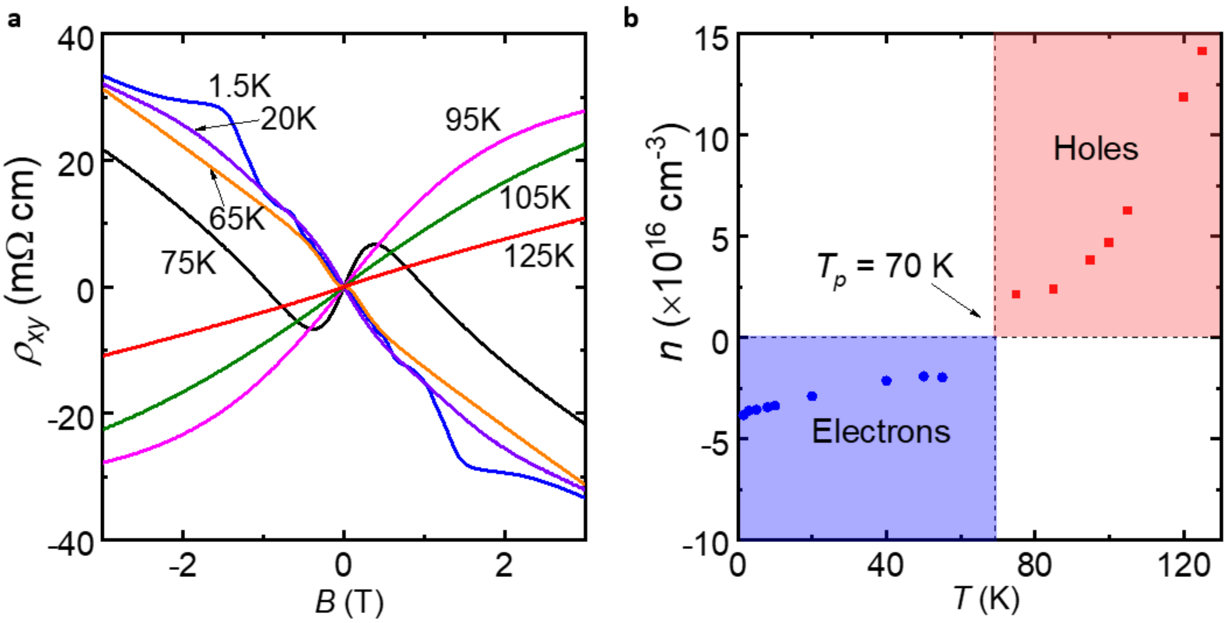

Supplementary Figure 3. a, Hall resistivity (ρ_{xy}) plotted as a function of magnetic field (**B**) at various temperatures (T) for the free-standing $HfTe_5$ sample F1. **b**, Charge carrier concentration extracted from the single band model fit of ρ_{xy} vs. **B** in a small range of -0.2 T < **B** < 0.2 T at various temperatures.

To better characterize the charge carriers in the as-grown CVT sample of $HfTe_5$, the Hall signal of the free-standing sample, F1, has been measured at temperatures below and above the peak temperature T_p as presented in Supplementary Figure 3a. Within the low magnetic field range (-0.2 T < **B** < 0.2 T), ρ_{xy} exhibits a linear magnetic field dependence at most of the measured temperatures except for that close to T_p, like T = 65 K (near T_p). The transport is dominated by electron- (hole-) like charge carriers at $T < T_p$ ($T > T_p$). A single band model can fit the ρ_{xy} vs. **B** very well for temperatures away from T_p with the extracted charge carrier densities given in Figure 3b. Our measured dependence of the carrier concentration vs. T is in good agreement with the Lifshitz transition observed in previous reports and it points to the chemical potential shifting from the valence band to the conduction band as the sample temperature is reduced.

IV. Comparison of different functionals for DFT calculations

We compare the experimental and calculated values of the lattice constants, volume, and band gap for different exchange-correlation functionals, as shown in Table S4. Due to the layered structure of $HfTe_5$, the consideration of van der Waals (vdW) correction is necessary. The optimized lattice constants of three functions with vdW corrections are close to experimental values: about 1% for optB86b-vdW and 2% for

DFT-D3 and SCAN-rvv10. If we consider the topological phase without strain, the optB86b-vdW and DFT-D3 methods will calculate that $HfTe_5$ is in the STI regime, as the previous first-principle studies showed [7,8]. However, as we discussed in the main text, SCAN meta-generalized gradient approximation can accurately treat short- to intermediate-range vdW interactions. If we use SCAN-rvv10 to calculate the same lattice constants as optB86b-vdW and DFT-D3, the band gap will increase from 62.1 meV to 106.4 meV for optB86b-vdW lattice constants and increase from 11.2 meV to 58.5 meV for optB86b-vdW lattice constants. The increase of the band gap shifts the phase transition point to the tensile strain side, leading to a WTI phase with a 3 meV band gap without strain.

Supplementary Table 4. Comparison between the experimental (EXP) and calculated values of lattice constants (a, b, c), volume (V), band gap at Γ point (E_g), and the topological insulating (TI) phase using different exchange-correlation functionals. Δa, Δb, Δc, ΔV represent the difference between the experimental and calculated values, i.e., $\Delta a = (a - a_{EXP})/a_{EXP} \times 100$. The topological insulator phases without strain calculated by different functionals are represented as STI and WTI.

	a (Å)	b (Å)	c (Å)	V (Å^3)	E_g (meV)	TI phase	Δa (%)	Δb (%)	Δc (%)	ΔV (%)
EXP	3.964	14.443	13.684	783.437	/	WTI	0	0	0	0
optB86b-vdw	3.980	14.574	13.750	797.552	62.1	STI	0.4	0.9	0.5	1.8
DFT-D3	3.995	14.700	13.603	798.913	11.2	STI	0.8	1.8	-0.6	2.0
SCAN-rvv10	3.981	14.754	13.579	797.660	3.0	WTI	0.4	2.2	-0.8	1.8
PBE	4.027	15.910	13.850	887.385	116.9	WTI	1.6	10.2	1.2	13.3

V. Band structure of $HfTe_5$ at zero strain

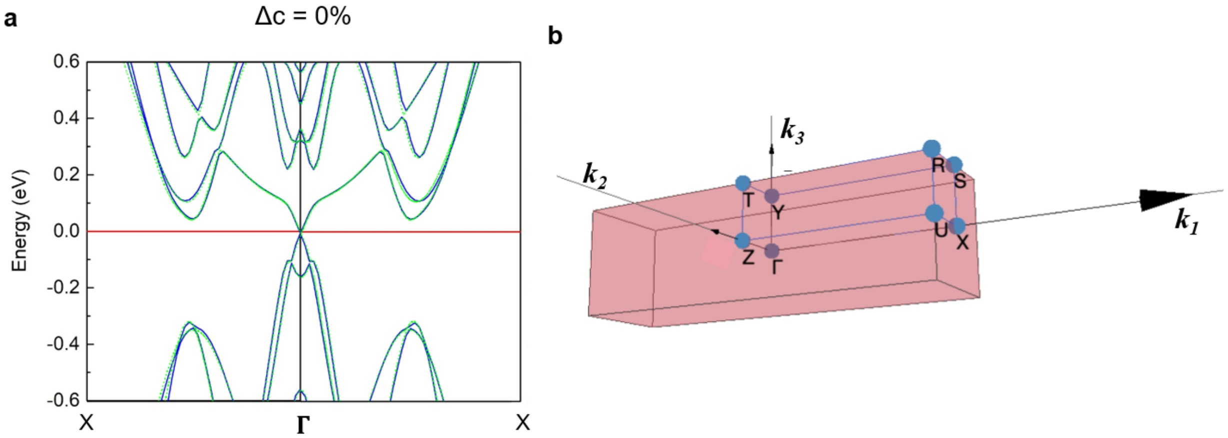

Supplementary Figure 4. a, Wannier fitted band structure of $HfTe_5$ without strain (green dashed lines) overlapped with the DFT band structures (blue lines). The red line represents the Fermi level. **b**, The reciprocal lattice of $HfTe_5$.

Supplementary Figure 4a shows the Wannier fitted band structures (green dashed lines) and the DFT band structures (blue lines). The perfect overlap between the Wannier and DFT band structures indicates that the Wannier functions we constructed with Te-p orbitals are high-quality for further calculations of topological properties. The reciprocal lattice of $HfTe_5$ is given in Supplementary Figure 4b.

VI. Wannier charge centers and $\mathbb{Z}_2$ indices under different strains

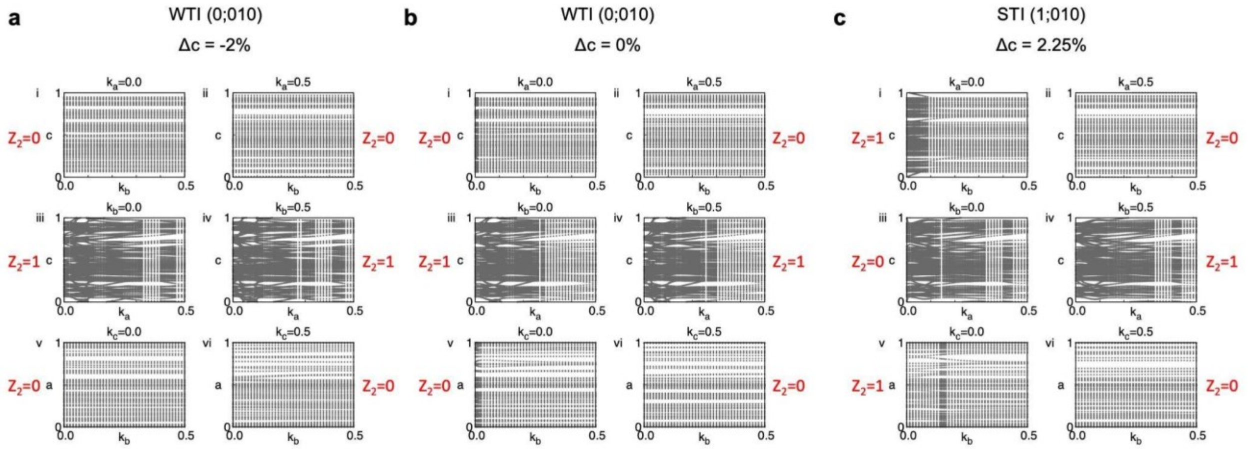

Supplementary Figure 5. Calculations of the Wannier charge centers on $HfTe_5$ at different strains **a**, for ϵ_C = -2%, **b,** for ϵ_C = 0 %, and **c**, for ϵ_C = 2.25%. For those applied strains the calculated $\mathbb{Z}_2$ indices (0;010), (0;010), and (1;010), are obtained respectively and labeled in each panel.

Next, we use WannierTools[9] to obtain the $\mathbb{Z}_2$ topological numbers in a 3D system by calculating the Wilson loop (Wannier charge center)[10] on six time-reversal invariant planes: $k_{1,2,3}$ = 0 and 0.5, respectively, as

shown in i-vi in Supplementary Figure 5a-c. The $k_{1,2,3}$ represents the reciprocal lattice vectors as shown in Supplementary Figure 4b. For each plane, the topological trivial or nontrivial nature can be determined by the even or odd number of crossings of the Wannier charge centers (WCC)[10], i.e., the even number of crossings means $\mathbb{Z}_2 = 0$ and the odd number of crossings means $\mathbb{Z}_2 = 1$, as shown in the red text at the side of each panel from i to vi from Supplementary Figures 5 a-c. Then, the strong and weak topological indices ($\nu_0;\nu_1\ \nu_2\ \nu_3$) of a 3D time-reversal-invariant (TRI) insulator can be determined from the 2D indices on the six TRI planes [11]. The strong topological index ν0 is nontrivial only if the Wannier charge centers on the $k_{1,2,3} = 0$ and 0.5 planes have different topological behavior. For compressive strain ϵ_C = -2% and zero strain ϵ_C = 0% as shown in Supplementary Figures 5a and 5b the $\mathbb{Z}_2$ indices are (0;010), implying $HfTe_5$ is in the WTI phase under those conditions. For tensile strain ϵ_C = 2.25% the $\mathbb{Z}_2$ indices are (1;010), implying $HfTe_5$ is in the STI phase under this strain condition.

VII. Band structures for various strains along the *c* axis

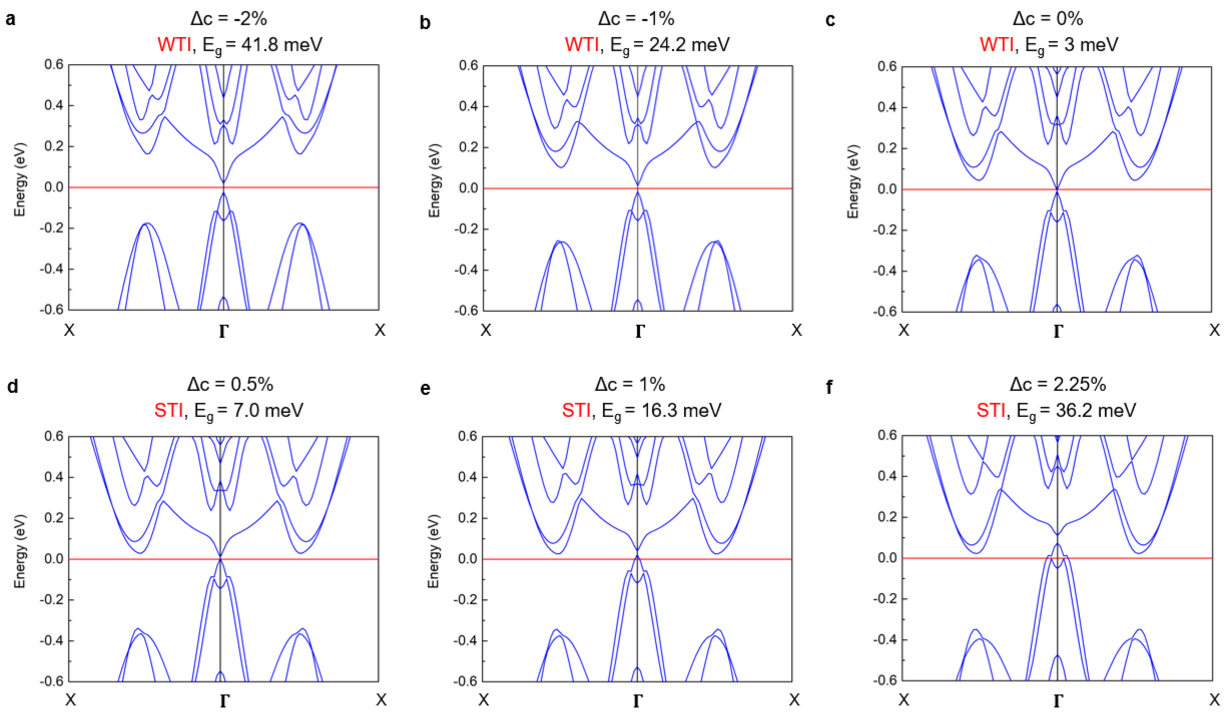

Supplementary Figure 6. a-f, The band structure of $HfTe_5$ under different strains along the *c* axis (ϵ_C) from -2% to 2.25%. The corresponding strain ϵ_C, the resulting topological phase, and the band gap at the Γ point are labeled in each panel.

The $HfTe_5$ band structure calculated for different strains along the *c* axis (ϵ_C) are shown in Supplementary Figure 6a-f, with strain varying from -2% to 2.25%. With the largest compressive strain (ϵ_C = -2%), the band gap at the Γ point can be increased to 41.8 meV. The system goes deep into the WTI phase, as the $\mathbb{Z}_2$

calculated in Supplementary Figure 5a. Similarly, with a larger tensile strain $\epsilon_C = 2.25\%$, the band gap at the Γ point can be increased to 36.2 meV. But in this case, $HfTe_5$ goes deep into the STI phase, as the $\mathbb{Z}_2$ calculated in Supplementary Figure 5c. Our calculations show that the gap closes and reopens with increasing ϵ_C as shown in Supplementary Figure 6, which is consistent with the phase diagram depicted in Figure 2d. Our simulations show that with increased strain a side band becomes important and could contribute with extra carriers, however we have not found experimental evidence of this extra band in our measurements. This may be due to a large effective mass band and potentially lower mobility. The band gap at the Γ point shows a V-shaped dependence with ϵ_C, as depicted in Supplementary Figure 7. Wannier calculations of surface states for the (010) top surface of $HfTe_5$ are shown in Supplementary Figure 8. For compressive strain -1% and 0% strain, there are no surface states within the bulk gap, as shown in Supplementary Figure 8 **a** & **b**. With increasing tensile strain, the topological surface states appear within the gap, as shown in Supplementary Figure 8 **c** and **d**. This is a direct evidence of the topological phase transition from WTI to STI under tensile strain.

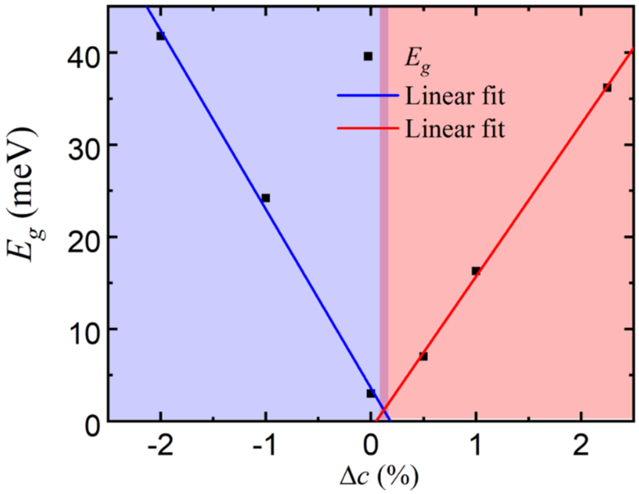

Supplementary Figure 7. The topological phase diagram of $HfTe_5$ based on our DFT calculations under different strains along the crystal c axis. The red and blue solid lines are linear to the band gap size obtained by the DFT calculations.

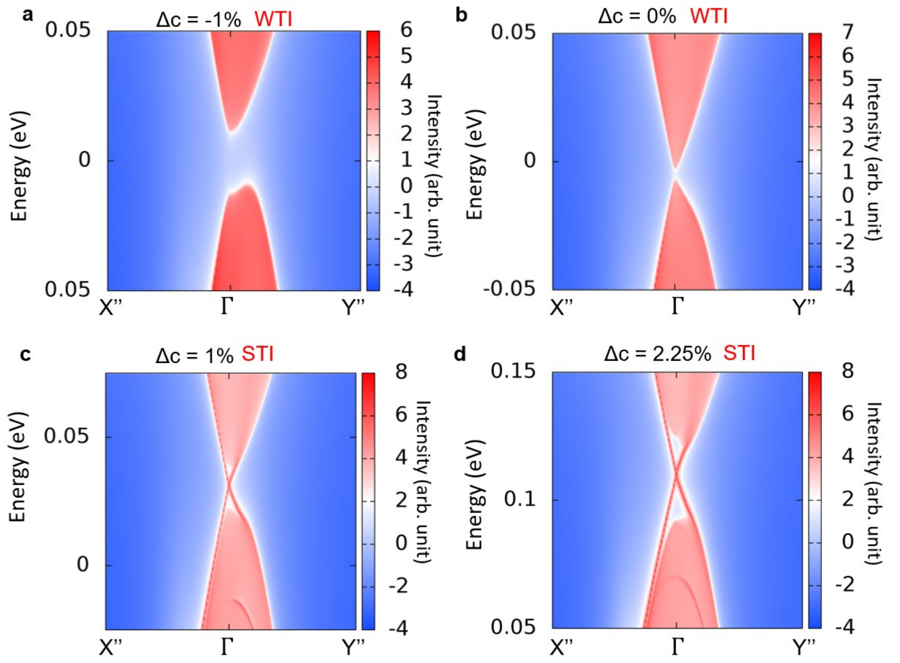

Supplementary Figure 8. The topological surface states spectrum of the top surface of $HfTe_5$ with strains of -1%, 0%, 1%, and 2.25% for **a-d**, respectively.

VIII. Application of small strain with a home-built single piezo-stack strain cell

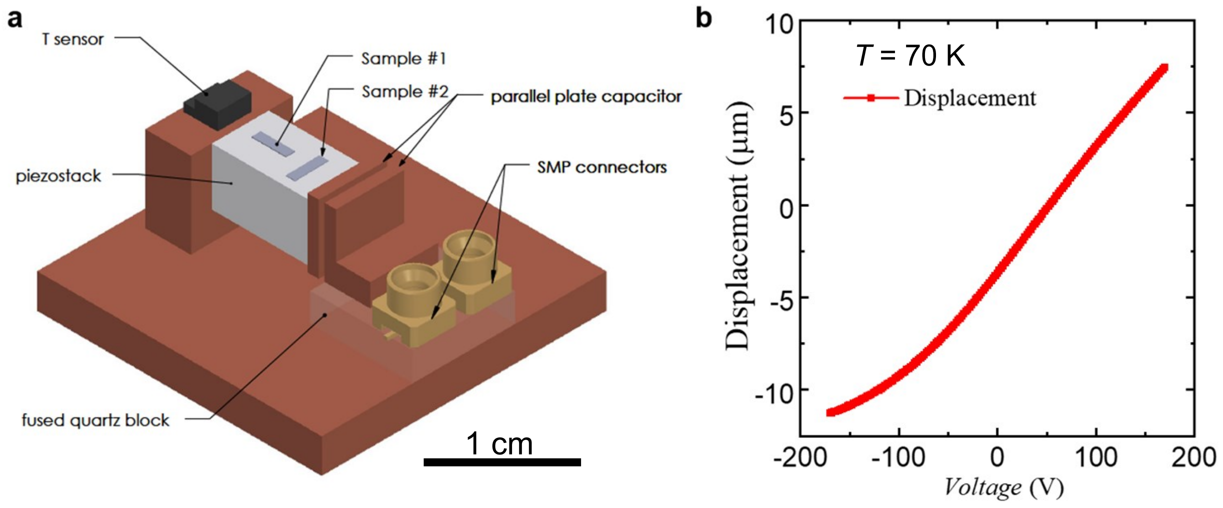

Supplementary Figure 9. a, Schematic of the single piezo stack strain cell. **b**, Displacement of the piezo stack as a function of voltage extracted by the capacitance measurement of the parallel plate capacitor used as a displacement sensor.

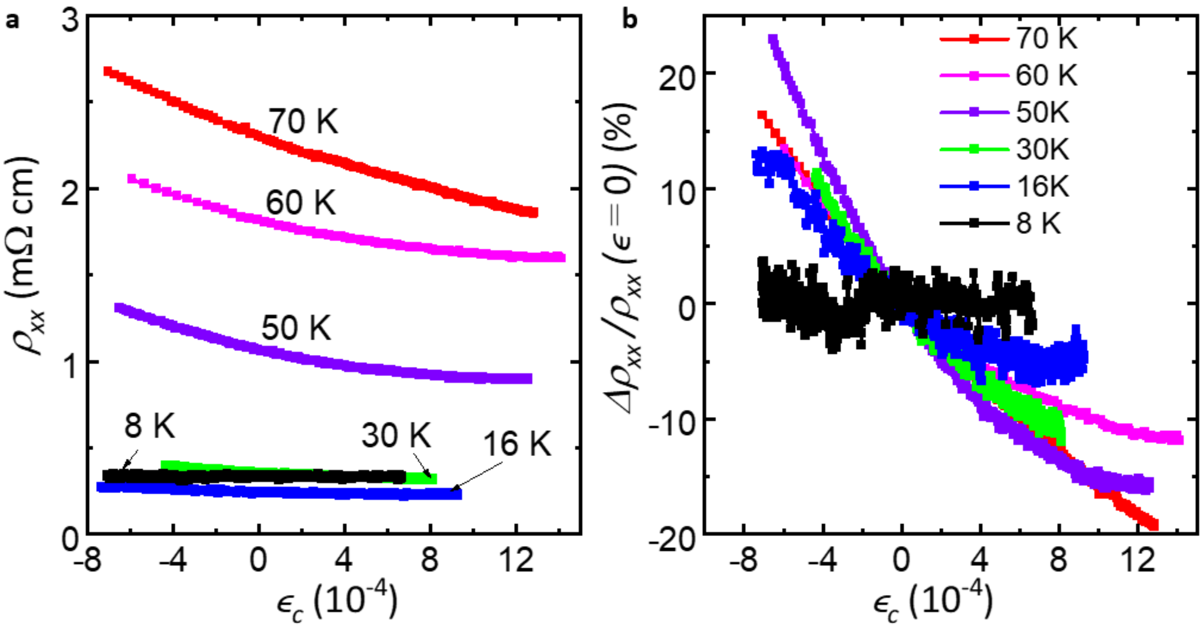

Supplementary Figure 10. **a**, Resistivity (ρ_{xx}) and **b,** piezoresistance, $\Delta\rho_{xx}/\rho_{xx}\ (\epsilon = 0) = \frac{\rho_{xx}(\epsilon)-\rho_{xx}(\epsilon=0)}{\rho_{xx}(\epsilon=0)}$, plotted as a function of strain for sample P1 at various temperatures. Note that the change in sample dimensions is not considered because of the extremely small strain.

Supplementary Figure 9 shows the schematic of a homebuilt single piezo-stack strain cell for applying small strain up to $\pm 0.1\%$ reliably on the van der Waals type single crystals of $HfTe_5$. Rectangular shapes on samples are glued on the top surface of the piezo stack with the long axis aligned either parallel or perpendicular to the poling direction of the piezo stack. The strain is calculated by $\epsilon = \frac{D}{L}$, where L is the effective length of the piezo stack and D is the displacement of the piezo stack. D is obtained by measuring the capacitance of the parallel plate capacitor that is attached to the piezo stack. Supplementary Figure 9b gives an exemplary change of D under a high voltage range at $T = 70$ K.

The piezo stack actuator used in this study is a PI Ceramic multilayer actuator (PICMA) which has a negative coefficient of thermal expansion in its length direction, which is approximately -2.5 ppm/K, provided by the manufacturer (PI Ceramic GmbH). As the sample *P1* is pasted on the piezo stack with the sample's *c* axis parallel to the poling direction, the sample experiences a non-negligible tensile strain at low temperatures. As depicted in Supplementary Figure 10, we notice that P1 shows a weakened strain dependence at $T \sim 16$ K, which indicates that the sample is at the DSM phase as shown in the topological phase diagram (Supplementary Figure 7). We observe the band gap closing at $T = 16$ K with $\epsilon_c < 0.1\%$ because of the combination of the applied strain and the strain caused by lowering the sample temperature

(~0.4±0.1 % for the lattice parameter change estimated from our single crystal XRD measurement, which agrees well with ~0.3% reported for $ZrTe_5$ by Zhang et al.[12]). At $T = 8$ K the ρ_{xx} slightly increases with the decrease of temperature and we believe this small increase is related to the beginning of gap reopening, as seen in sample B1 at ϵ_1. The temperature dependence of the ρ_{xx} for the free-standing samples (samples F1 and F2) are different from the sample pasted on the piezo actuator. For the free-standing sample, sample F1, the ρ_{xx} reduces continuously for $T < 70$ K, while for P1, the ρ_{xx} starts to increase for $T < 16$ K. We believe the main difference is related to the negative thermal expansion coefficient of the piezo stack of -2.5 ppm/K which corresponds to 0.075% of expansion by cooling down to $T = 1.5$ K. As thus, an effective tensile strain of ~ 0.475±0.1 % is applied along the c axis for P1. Supplementary Figure 10b shows the dependence of ρ_{xx} with strain and it demonstrates that positive strain (ϵ_C) becomes less effective in modulating the band gap for $T < 16$ K.

IX. Application of large strain with the home-built bending apparatus

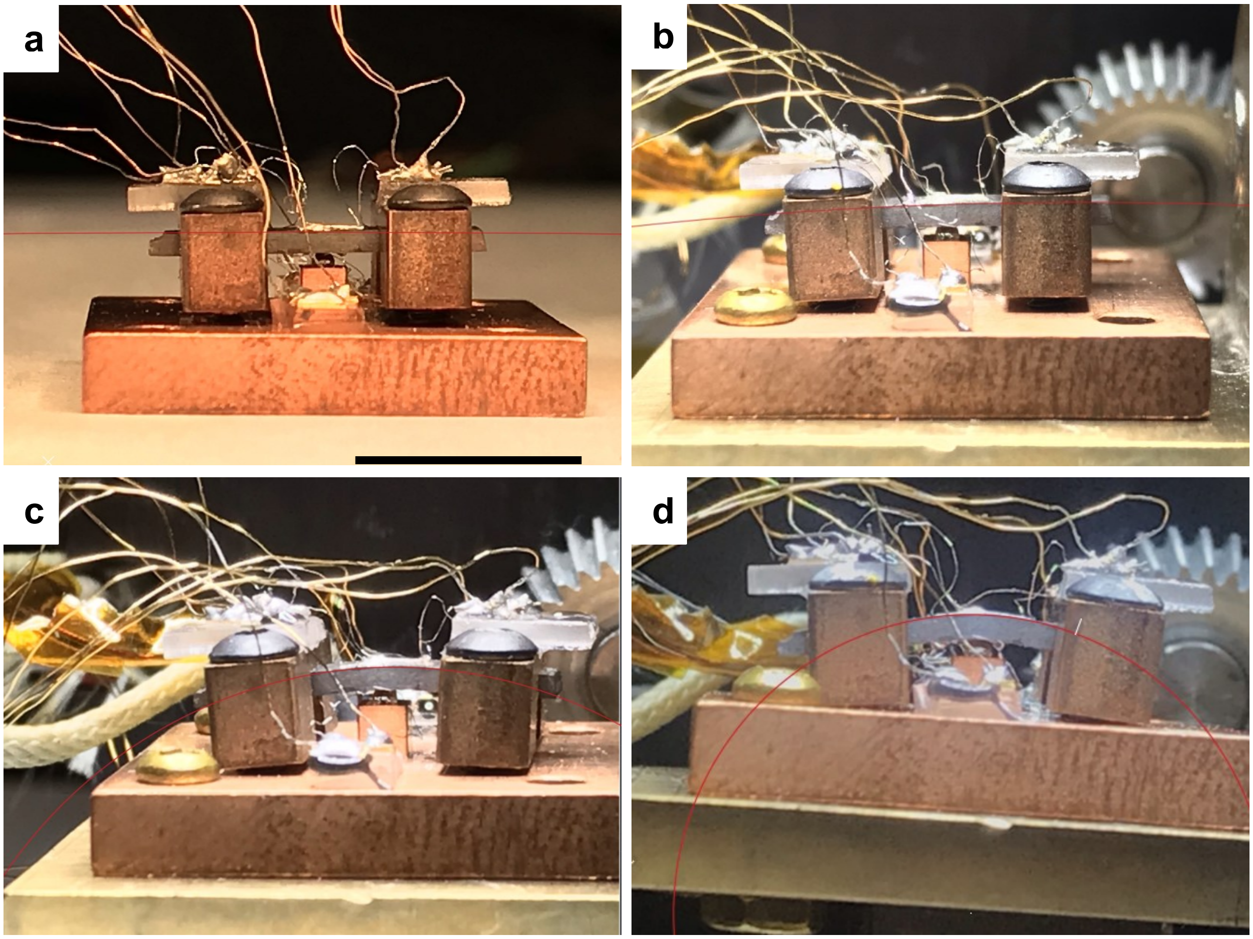

Supplementary Figure 11. a-d, Optical images of the bending strain cell with increasing strains from ϵ_0 to ϵ_3. The scale bar represents 1 cm. The red line is the fit to a circle with three points on the circumference. The resulting strain values for different strain cases are as follows $\epsilon_0 = 0.04\%$; $\epsilon_1 = 0.26\%$; $\epsilon_2 = 2.3\%$; $\epsilon_3 = 4.5\%$.

Supplementary Figure 11 shows the optical images of the bending strain cell in the cases of high bending strain for this study. The bending radius of the beam is estimated by carefully selecting three points around the middle of the beam's top edge and fitting them to the circumference of a circle, which is done with AutoCAD 2022 Three Points Circle Command. The strain is thus calculated by $\epsilon_i = \frac{t}{2R_i}$, where t is the thickness of the beam and R_i is the bending radius for i^{th} strain. To ensure a consistent and robust bond between the sample and the top surface of the Ti substrate beam, we have employed epoxy with a lower viscosity. When the samples are placed on top of the epoxy layer, the epoxy will go on to the side surfaces of the samples. Once the epoxy is cured, it takes on a structural form as simplified in Supplementary Figure 12b instead of the case that the epoxy layer is only at the bottom of the sample as shown in Supplementary Figure 12a. The strain transmitted onto our sample is simulated for the medium high strain case. It shows such a structural arrangement can effectively facilitate the uniform application of significant stress across the sample.

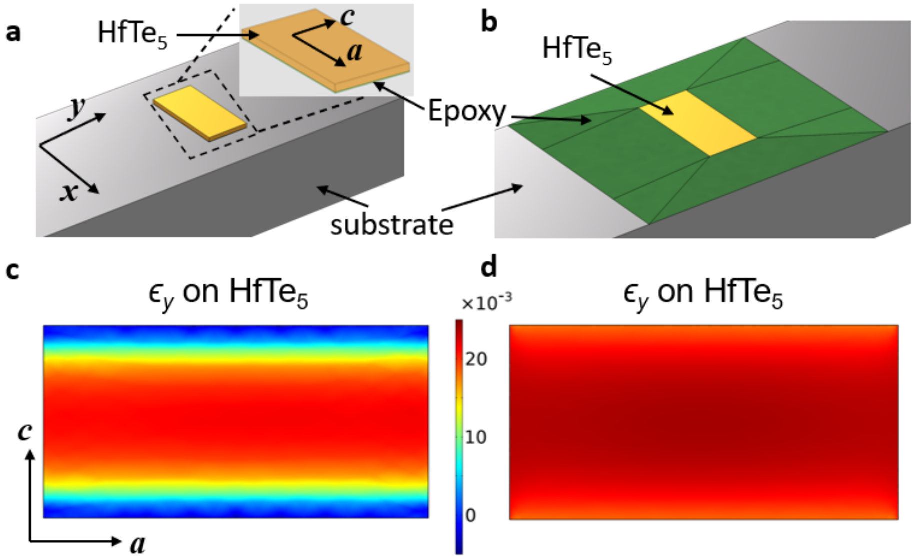

Supplementary Figure 12. COMSOL simulation of strain transmitted onto $HfTe_5$. **a,** Schematic of a $HfTe_5$ sample affixed to the titanium bending beam by a thin bottom layer of epoxy. **b**, Schematic of the experimental situation where the sample side surfaces are wrapped by the epoxy after curing. **c** & **d,** describe strain ϵ_y transmitted to the $HfTe_5$ sample for cases **a** and **b**, respectively.

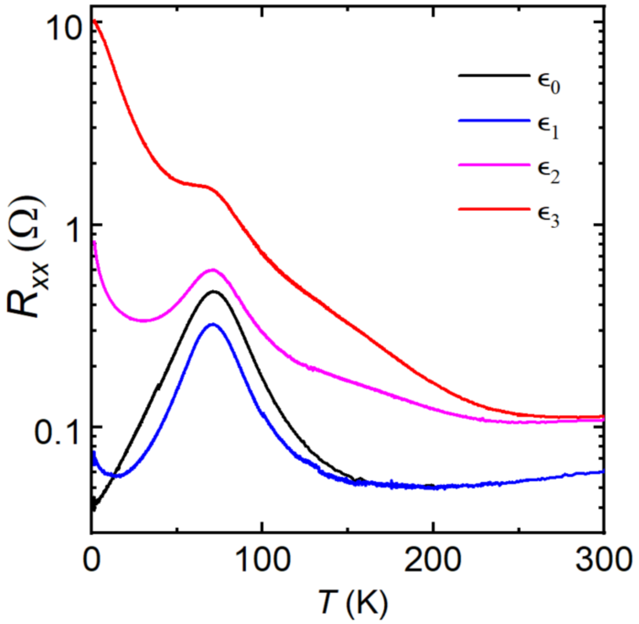

Supplementary Figure 13. 4-probe resistance (R_{xx}) vs. temperature (T) of sample B1 glued on the bending station measured under different strains.

Raw data of the 4-probe resistance as a function of temperature data for the measurement of sample B1 under different strains is provided in Supplementary Figure 13 plotted in a log-linear scale to show the clear change of the R_{xx} vs T. The SdH oscillations are measured at various temperatures for different strain cases for the analysis of cyclotron effective mass (m*), as shown in Supplementary Figure 14. With increased strain, m* tends to become smaller. At ϵ_2, we see a significant reduction (~ 22%) in effective mass, which may be attributed to the linear Dirac cone dispersion of the surface electrons. However, we acknowledge that this may not exclusively result from TSS dominant conduction.

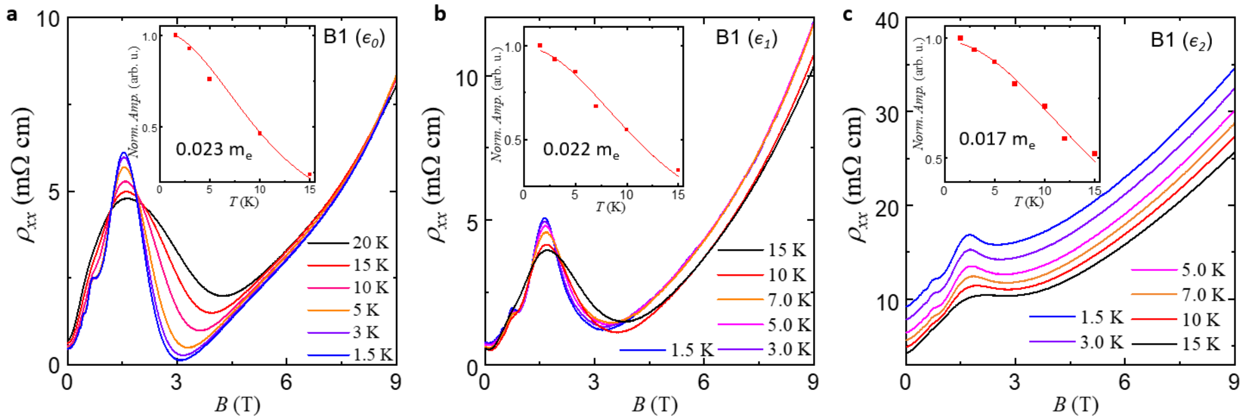

Supplementary Figure 14. Analysis of cyclotron effective mass of the sample B1 under strain ϵ_0, ϵ_1, and ϵ_2. ρ_{xx} as a function of the magnetic field at various temperatures under strain ϵ_0 in **a**, strain ϵ_1 in **b**, and strain ϵ_2 in **c**. The insets are the fitting to the thermal damping term of the LK equation.

We have employed a fitting procedure to model the temperature dependence of resistance (R vs. T) under high strain, ϵ ~ 4.5 %. To more quantitatively extract the surface contribution to the total (sheet) conductance (G^{tot}), we fit our $R_{sh}(T)$ data to a simple model used in Ref. [45], where the total conductance $G^{tot} = 1/R_{sh}(T)$ is the parallel sum of the thermally activated bulk conductance which contributes with G^{bulk}(T) = $t(\rho_{b0}e^{\Delta/kT})^{-1}$, where t is the thickness of the sample, k is the Boltzmann constant with the fitting parameters being ρ_{b0}, the high temperature bulk resistivity, and Δ, the activation energy, and a metallic surface conductance $G^{sur}(T) = (R_{sh0}+\mathrm{A}T)^{-1}$, where the fitting parameters are R_{sh0}, representing the low-T residual resistance (due to impurity scattering), and A, reflecting the electron-phonon scattering. The fitting results consistently yield 100% surface conduction at low temperatures as shown in the main text and the Supplementary Fig. 15c. Particularly, for B2 under strain ϵ ~ 4.5 % we obtain Δ = 50.6 meV, R_{sh0} = 0.01 Ω/sq and A = 0.02285 Ω/K. This result indicates 100% surface conduction for temperatures below 50 K for B2, while for B1 for temperatures below 10 K. The ratio of the surface conductance contribution to total conductance, G^{sur}/G^{tot}, is obtained from the fitted G^{sur} divided by the experimental G^{tot} data.

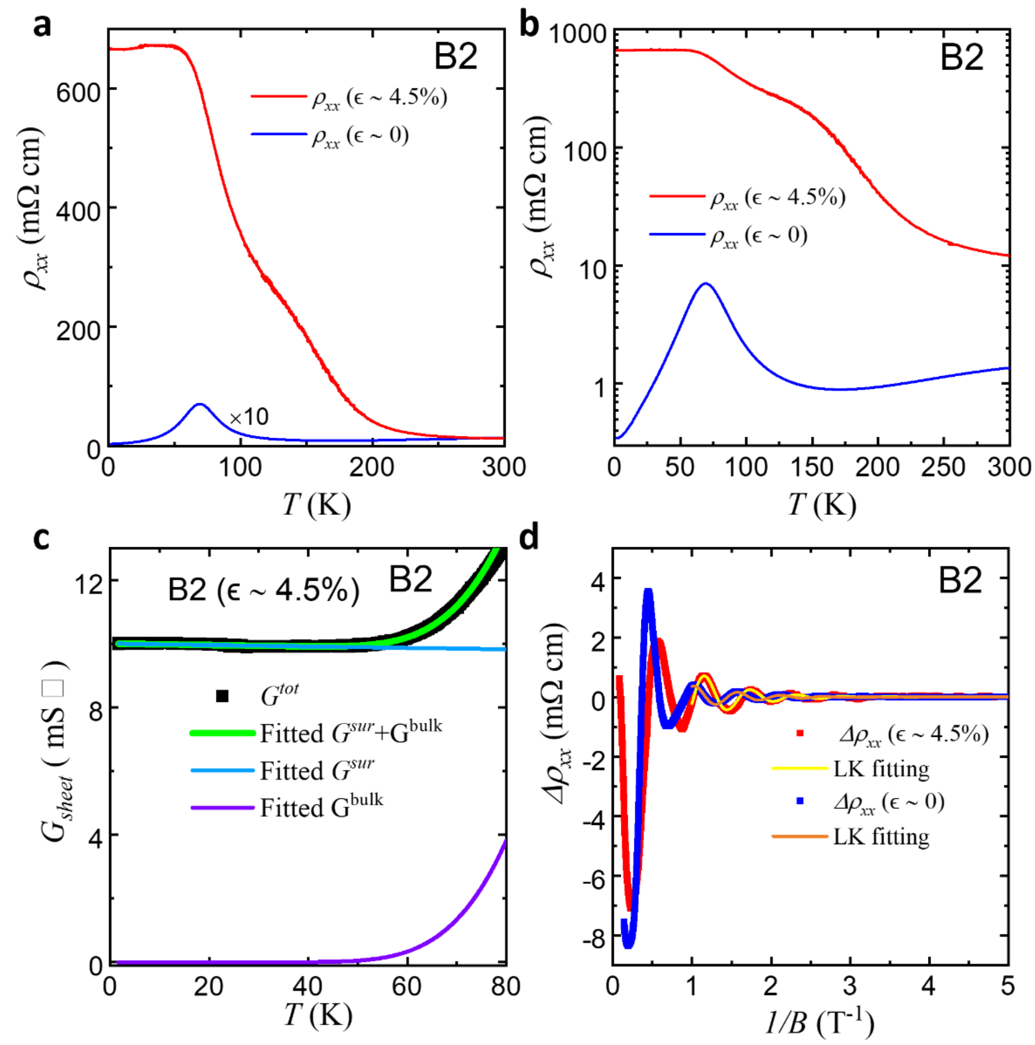

Supplementary Figure 15. Comparison of the electronic transport behaviors under zero strain ($\epsilon \sim 0$) and under a large strain ($\epsilon \sim 4.5$ %) for sample B2. **a.** ρ_{xx} as a function of temperature under $\epsilon \sim 0$ (blue) and $\epsilon \sim 4.5\%$ (red) plotted in linear scale. **b.** same plot as in **a**, shown on a log-log scale for clarity. **c.** Fitting of low temperature sheet conductivity with contributions from both surface and bulk conduction for sample B2 under strain $\epsilon \sim 4.5$ %. **d.** SdH oscillations under a perpendicular magnetic field plotted against the inverse magnetic field (1/**B**) for strain $\epsilon \sim 0$ (blue) and strain $\epsilon \sim 4.5\%$ (red). The bright lines are fittings to the LK equation, resulting $\gamma = 0.11$ and -0.0053 for $\epsilon \sim 0$ and $\epsilon \sim 4.5\%$, respectively.

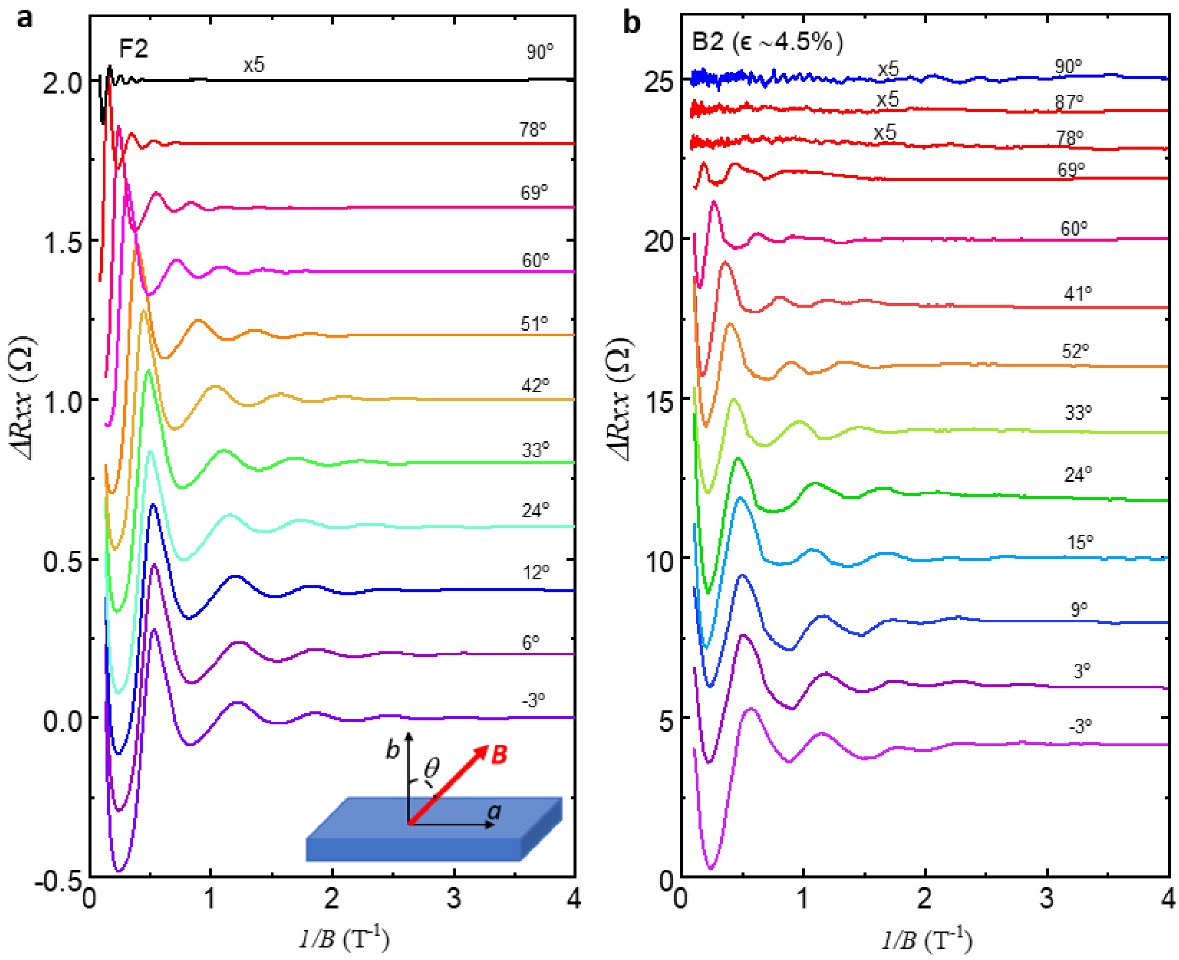

Supplementary Figure 16. Comparison of the Fermi surface dimensionality through the measurement of angular dependence of the SdH oscillations. The oscillatory component of resistance, ΔR_{xx} vs. 1/**B** measured under different field orientation angles θ for a free-standing sample (sample F2) in **a** and sample B2 under a large strain ($\epsilon \sim 4.5$ %) in **b**. The angle θ is defined as the polar angle between the magnetic field and the sample's b axis, as shown in the inset of **a**. For $\theta > 70^{\circ}$, the SdH oscillations are no longer observed in B2 ($\epsilon \sim 4.5$ %).

Supplementary Table 5. A summary of oscillation frequency, F and phase shift, γ extracted from the LK fitting of the SdH oscillations at $T = 1.5$ K under a perpendicular magnetic field for different samples.

**Samples**	**F1**	**F2**	**B1**			**B2**	
			ϵ_0	ϵ_1	ϵ_2	ϵ_0	$\epsilon \sim 4.5\%$
F (T)	0.97	1.52	1.21	1.32	1.40	1.82	1.72
γ	0.14	0.13	0.12	0.13	-0.0060	0.11	-0.0053

We would like to note that we have not observed an abrupt change in the SdH oscillations with strain. This may be due to the small and similar sizes of the Fermi surfaces from the bulk and surface states, as suggested by our DFT calculations and as indicated by our measured small oscillating frequency of ~ 1 T and the light cyclotron effective masses measured at all strain levels. Also, we have not observed any beating patterns in the quantum oscillations or other signatures of the coexistence of bulk and surface states carriers. This may be due to the 100% surface contribution we observed in the ρ_{xx} vs. T with an insulating bulk at low temperatures and high strains.

Supplementary References

1. APEX2 Version 2014.11-0, Bruker AXS, Inc.; Madison, WI 2014.
2. SAINT Version 8.34a, Bruker AXS, Inc.; Madison, WI 2013.
3. Sheldrick, G. M. SADABS, Version 2014/5, Bruker AXS, Inc.; Madison, WI 2014.
4. Sheldrick, G. M. SHELXTL, Version 2014/7, Bruker AXS, Inc.; Madison, WI 2014.
5. International Tables for Crystallography 1992, Vol. C., Dordrecht: Kluwer Academic Publishers.
6. Furuseth, S., Brattås, L. & Kjekshus, A. The Crystal Structure of HfTe5. *Acta Chem. Scand.* **27**, 2367–2374 (1973).
7. Weng, H., Dai, X. & Fang, Z. Transition-Metal Pentatelluride $ZrTe_5$ and $HfTe_5$: A Paradigm for Large-Gap Quantum Spin Hall Insulators. *Phys. Rev. X* **4**, 011002 (2014).
8. Fan, Z., Liang, Q.-F., Chen, Y. B., Yao, S.-H. & Zhou, J. Transition between strong and weak topological insulator in $ZrTe_5$ and $HfTe_5$. *Sci. Rep.* **7**, 45667 (2017).
9. Wu, Q., Zhang, S., Song, H.-F., Troyer, M. & Soluyanov, A. A. WannierTools: An open-source software package for novel topological materials. *Comput. Phys. Commun.* **224**, 405–416 (2018).
10. Soluyanov, A. A. & Vanderbilt, D. Wannier representation of $\mathbb{Z}_2$ topological insulators. *Phys. Rev. B Condens. Matter* **83**, 035108 (2011).
11. Taherinejad, M., Garrity, K. F. & Vanderbilt, D. Wannier center sheets in topological insulators. *Phys. Rev. B Condens. Matter* **89**, 115102 (2014).
12. Zhang, Y. *et al.* Electronic evidence of temperature-induced Lifshitz transition and topological nature in $ZrTe_5$. *Nat. Commun.* **8**, 15512 (2017).
